# Scientometric Analysis of Dental Implant Research over the Past 10 Years and Future Research Trends

**DOI:** 10.1155/2021/6634055

**Published:** 2021-04-13

**Authors:** Xin Huang, Jin Bai, Xu Liu, Zhaosong Meng, Yuli Shang, Tiejun Jiao, Gang Chen, Jiayin Deng

**Affiliations:** ^1^Department of Oral and Maxillofacial Surgery, Stomatological Hospital of Tianjin Medical University, Tianjin, China; ^2^Department of Oral Implantology, Stomatological Hospital of Tianjin Medical University, Tianjin, China; ^3^Department of Periodontics, Stomatological Hospital of Tianjin Medical University, Tianjin, China

## Abstract

**Background:**

We conducted a bibliometrics analysis to explore the recent trends in dental implant research which could help researchers have a clear grasp of the relevant research hotspots and prospects. *Material and Methods*. Altogether, 15,770 articles on dental implants, from January 1, 2010, to October 31, 2019, were selected from the Web of Science Core Collection. We used BICOMB software to extract the high-frequency MeSH terms and construct binary and coword matrices. gCLUTO software was used for biclustering and visual analysis, Ucinet 6 software for social network analysis, SCIMAT software for strategic diagram building, Citespace 5.5 software to form timeline visualization, and VOSviewer software, eventually, for bibliometrics cocitation network.

**Results:**

Altogether, 72 high-frequency keywords were extracted from the selected articles and 4 clusters and 7 subcategories were identified through biclustering analysis in the dental implant research field. The use of the strategic diagram also enabled us to find the research hotspot and development trends.

**Conclusions:**

The survival rate of dental implants and subsequent restoration have always been the core focus of research. Sinus floor elevation and guided bone regeneration are worthy of constant exploration owing to their reliability. With continuous improvement in technology, immediate loading could become a future research hot spot.

## 1. Background

Oral implantology has been developing into a specialized field since the 1930s under the initiative of Formiggini (the father of modern implantology in Egypt) and Zepponi. However, compared with clinical applications, the situation of theoretical study in the field had remained in its infancy for many years, which changed in 1950, when Professor Branemark from the University of Gothenburg in Sweden put forth the osseointegration theory [1], and led to a huge development in oral implantology.

A dental implant is a device fitted into the jawbone to transmit bite force. Achieving osseointegration is the biological prerequisite for implant success. Over the years, researchers have extensively experimented with implant materials to ensure both biocompatibility and good biological properties. In the early stages of clinical research, Branemark used titanium screw-type implants with a machined surface [1, 2]. On the other hand, the Schroeder International Team used implants with a titanium plasma-sprayed surface [3–5]. Since then, more and more types of implant materials have also begun to be used in clinical applications. Implant system materials have evolved from containing aluminum oxide and titanium aluminum vanadium alloy to being made of pure titanium [6–8].

As the global population continues to expand and age, researchers are continuing to study implant survival rates. In terms of the implant itself, both the type and diameter of the implant surface may affect failure rate, while smoking and taking antidepressants can also affect failure rates in other respects [9, 10]. Besides, dental implant research on diabetics is also growing, as the incidence of diabetes increases year by year. Glucose control levels are being increasingly recognized as the golden standard for achieving good osseointegration and improving the success rate of dental implants. Evidence has also suggested that prophylactic use of antibiotics can increase the implant success rate of patients with diabetes to 96.47% [11].

The concepts and materials underlying the development of dental implants are improving and being further developed. Therefore, research on dental implants can be assessed through a coword analysis of the literature by using the bibliometrics analysis method [12] to point out trends in dental implant development and hotspots in the field for other researchers. In the analysis of cowords, if two keywords simultaneously appear frequently, it can be that there is a close relationship between them. On this basis, a double-clustering analysis on the subject words can be performed, with the rows as well as the columns being clustered at once in the matrix [13, 14]. Bibliometrics cocite network mapping and timeline visualization have been used for revealing the links between articles and time nodes.

In this study, we sought to look for potential and promising dental implant research areas using a scientometric analysis approach and to reveal any latent value in previous research. While dental implants have been overwhelmingly developed in recent years, an overview of the evolution and use of this technique is still lacking. Using the coword analysis, we can provide the most intuitive and scientific snapshot of the research directions and recent trends in the field of dental implants.

## 2. Methods and Materials

### 2.1. Data Collection

We searched the Web of Science Core Collection (WOSCC) for relevant articles. The Web of Science is published by Thomson Reuters and contains more than 5900 journal abstracts and citations, covering various fields of medicine and the natural sciences. The collection criteria were as follows: articles with the theme of dental implants published from January 1, 2010, to October 31, 2019. Altogether, 15,770 articles were found. The article, title, keywords, author information, publication time, and references were then exported and downloaded in TXT format.

### 2.2. Data Extraction and Analysis

Bibliographic items cooccurrence matrix builder (BICOMB) is a basic software tool that was developed by Lei at China Medical University for text mining [15]. In this study, BICOMB was used to filter data and construct matrices. When BICOMB determines keyword frequency, it can be executed for data cleaning by grouping different expressions with the same meaning into one keyword: dental implant and dental implants and Dental implant and Dental Implant with different singular and plural forms, capitalization, and abbreviation and with or without spaces. High-frequency keywords were defined as appearing more than 60 times. Subsequently, high-frequency keywords, a dental implant binary matrix, and cooccurrence matrix of keywords were created. Next, we performed cooccurrence analysis and double-clustering analysis on the filtered data and matrix. Cooccurrence analysis uses the cooccurrence of vocabulary and noun phrases in the literature set to determine the relationship between the keywords in the field. It is generally believed that the more times a vocabulary pair appears in the same document, the thicker the connection is between the two keywords. Ucinet 6 software, which was designed by Stephen Borgatti and colleagues from the University of California Irvine, allows performing a cooccurrence analysis for social network analysis (SNA) [16]. Double-clustering analysis cluster data in both the row and the column directions of the matrix. In the resulting cluster, the data are related to each other in both the rows and the columns. We used gCLUTO software to generate mountain peak map and then to summarize and analyze the relationship between each category of dental implants. SCIMAT software was used for strategic coordinate diagram construction [17]. Further, we determined the subcategories of dental implants.

### 2.3. Cocitation Analysis

Cocitation analysis is a quantitative research method that was developed by Henry Small for scientific evaluation, academic analysis, and information retrieval [18]. Small and Griffith believe that cocited article clusters characterize currently active research areas [19]. We thus used Citespace 5.5 software, a knowledge mapping tool developed by Chen [20, 21], based on JAVA for cocitation analysis. The role of prominent points can be verified by consulting the literature or experts in the field. VOSviewer software was then used for cocitation analysis and the creation of a network map based on bibliographic data. The analysis considered all authors and their affiliations as states in the articles. VOSviewer was developed by Eck and Waltman at Leiden University's Centre for Science and Technology Studies [22].

## 3. Results

Altogether, 15,770 articles on dental implants dated between January 1, 2010, and October 31, 2019, were searched and extracted from the Web of Science. Seventy-two high-frequency keywords were extracted from the selected articles using BICOMB software (Table 1) and subsequently used to build binary matrices (Table 2) and co-occurrence matrices (Table 3).

### 3.1. Social Network

**Figure 1:**
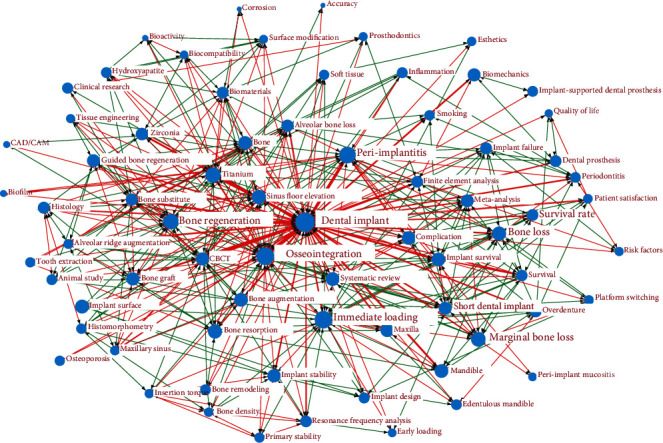
Social network diagram on dental implant keywords.

Ucinet 6 software was used to perform a cooccurrence analysis on a cooccurrence matrix. Furthermore, a social network analysis (SNA) was constructed (Figure 1) to analyze the subject and structure of dental implants. In the same research articles, the simultaneous appearance of different keywords reflects relevance and links between keywords. The higher the frequency of simultaneous keyword occurrence, the greater the relevance to the keywords and the closer to the center of the social network diagram. For example, dental implant, osseointegration, titanium, peri-implants, survival rate, bone-regeneration, CBCT, sinus floor elevation, immediate loading, and zirconia have the greatest centrality and relevance, which have also become significant research foci of great value. The thicker and redder the lines between the subject words, the stronger the connection between them (e.g., sinus floor elevation and dental implant, CBCT and dental implant, osseointegration, and dental implant). However, green and regular lines represent relatively weak connections compared to the red lines.

### 3.2. Peak Map

**Figure 2:**
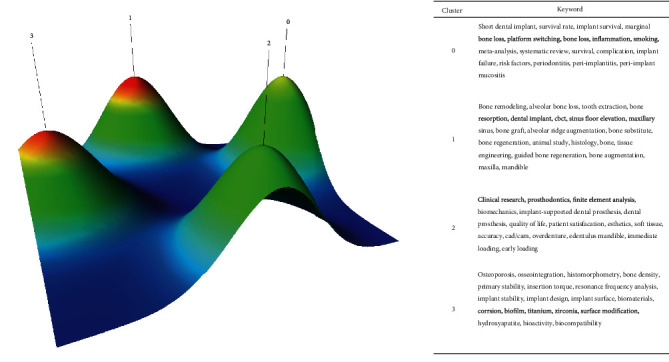
Peak visualization biclustering of highly frequent keywords in articles of dental implants.

The 72 high-frequency keywords were divided into 4 clusters (numbered from 0 to 3) after calculating all the possibilities of clustering with minimum standard deviation and minimum rank sum. A peak map was then generated (Figure 2). The distance between peaks and the volume, height, and color of the peaks were used to describe the related clusters. The distance indicates the relative similarity of the clusters. The peak volume is directly proportional to the number of keywords in each cluster. The higher the peak, the closer the similarity of the internal distribution in the cluster. In addition, the color of the peaks quantifies deviation in the cluster, with red standing for low deviation and blue for high deviation. Hence, we classified the 72 high-frequency keywords in dental implant research over the past 10 years into 4 clusters based on the attention that the high-frequency keywords received themselves and in relation to each other. Furthermore, these four major categories were further divided into the following 7 subcategories: (1) factors affecting implant osseointegration (Cluster 3), (2) preparation and treatment of dental implants (Cluster 1), (3) early stability of dental implants (Cluster 3), (4) implant surgery complications and risk factors (Cluster 0), (5) treatment after dental implantation (prosthesis) (Cluster 2), (6) patient satisfaction after surgery (Cluster 2), and (7) survival rate of dental implants (Cluster 0).

### 3.3. Strategic Diagram for Dental Implant Research

**Figure 3:**
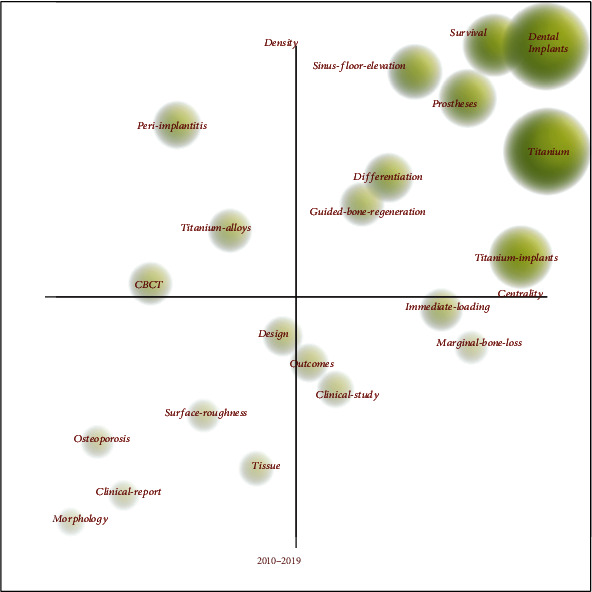
Strategic diagram for keywords in recent articles on dental implants.

Hotspots correspond to the keywords with strong internal and external cohesion (density and center, respectively) in Figure 3 (e.g., in the first quadrant of survival, sinus floor elevation, prostheses, titanium, differentiation, and guided bone regeneration). These hotspots are at the core of research, with a high degree of received attention and maturity. The keywords contained in the second quadrant refer to those areas that have a high degree of development maturity but have a low level of received attention, such as peri-implantitis and CBCT. The keywords in the third quadrant are considered insufficient in both density and centrality, representing poor maturity. Meanwhile, keywords in the fourth quadrant are hardly discussed in the field but have a probability of becoming future research hotspots. The larger the volume of the sphere, the larger the number of articles with the term.

### 3.4. Bibliometrics Cocitation Network

**Figure 4:**
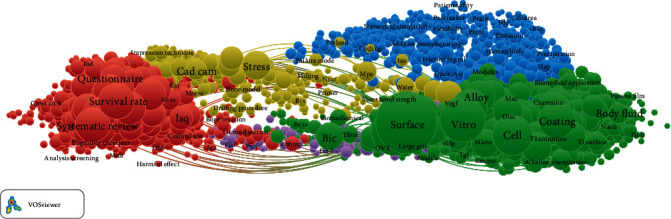
Cocitation network of articles on dental implants.

In the dental implant cocitation network, each keyword was represented by a circle (Figure 4). The size of the circle represents the number of articles, and different colors represent various research directions. Journals were roughly divided into four major categories by Citespace, which also confirmed the accuracy of the classification of the peak map appropriately. The red portion of the network indicates issues with the implant survival rate. The yellow portion represents the second-stage restoration of the implant and different types of scientific analysis methods. The blue portion represents the treatment after dental implant surgery. The green portion represents the treatment of the dental implant surface and the type of material. The distance between two articles in the visualization roughly represents the relevance of these articles in terms of cocitation links.

### 3.5. Timeline Visualization

**Figure 5:**
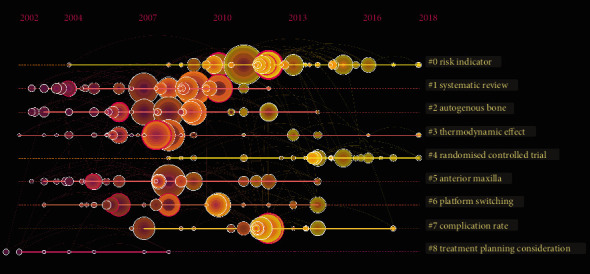
Timeline visualization of research trends in dental implants.

The timeline view shows changes in research trends over a specific time node, with the nodes of the cluster sharing a horizontal line (Figure 5). The top of the view represents the time when the article was published, going from 2010 to 2019. The size of each node represents the number of nodes, with the larger the nodes, the greater their number and importance (e.g., Lang NP (2011) and Jung RE (2012)). In addition, the timeline view shows the temporal characteristics of periodic implants in the field of dental implants. As can be seen in Figure 5, the development of #2 (autogenous bone) occurred first, indicating that early research on dental implants focused on the causes of autologous bone derived bone augmentation methods. While #0 (risk indicator) and #7 (complication rate) showed late development, it may be that the increase of implant quantity in recent years has brought about the increase in failure rates. More scholars focus on the factors influencing the success of implants, highlighting the need to pay more attention to potential risks and complications.

## 4. Discussion

The development of dental implantology has made considerable progress. It has developed rapidly within 40 years. Dental implantation has revolutionized the concept of traditional dental prostheses, which has made significant changes in the concept and content of dental prosthetics. As a method, the implants show a clear advantage in clinical application. With the development of technology in the field of stomatology, especially in the field of dental implantology, the number and content of professional scientific literature and academic journals in this field continue to grow, and the interconnection between individual authors has become closer. A systematic analysis of this increase in scientific publication is needed so that the academic community can quantify research results and research impact. In this regard, bibliometrics indicators are useful and objective tools for evaluating the results of scientific activities. Through the use of bibliometrics tools, we can determine the research hotspots, research frontiers, the most fruitful authors, research centers, and the cooperation mode among published authors in this field regarding implant.

We conducted a cooccurrence and cocitation analysis of the literature on dental implant research in the past 10 years using different bibliometrics tools. To ensure the accuracy of the data, we tried to construct a variety of subject maps, organizing and analyzing them as much as possible. When multiple subject maps showed great research interest in some categories, we considered that the high-frequency keywords in this category had remarkable centrality and potential value in the field. These high-frequency keywords in the social network were consistent with the information obtained in the strategic coordinate diagram. For example, guided bone regeneration (GBR) was both a hotspot in the strategic coordinate and a direct link to dental implants. It has thus been suggested that GBR is a necessary step to solve the clinical problem linked to insufficient bone mass for implantation [23].

In the strategic map, the keywords in the first quadrant: survival (Cluster 0), sinus floor elevation (Cluster 1), prostheses (Cluster 2), differentiation (Cluster 3), guided bone regeneration (Cluster 1), and titanium (Cluster 3), showed a high degree of research attention and potential in the field of dental implants.

In quadrant I, survival (Cluster 0) had a high degree of received attention and outstanding research potential. The survival rate of implants has always been the most important issue for the application of implants to the clinic. The factors affecting implant survival generally depend on both the implant and the patient. Thus, the material of the implants as well as the surface morphology and treatment can influence survival rate [24]. Goyal and Priyanka [25] suggested that the surface area of the implant could be increased by raising the roughness of the implant, thereby promoting cell migration and osseointegration with the implant. Chappuis et al. [26] suggested that sandblasting and acid etching on the surface of titanium implants have the greatest effect on the formation of osseointegration. Surface roughness of dental implants may also affect osteoblast differentiation [27]. With the rapid development of digital medicine in recent years, surgical navigation and virtual reality surgery will likely become the future mainstream trends of implant surgery, because digital surgery can evaluate the results of guided surgery based on the survival rate, operation accuracy, and surgical complications of the implant [28]. Patients' factors such as bruxism, smoking, and intake of antidepressants are thought to directly affect the survival rate of implants [29, 30]. Some high-risk factors like diabetes are considered to present a safe level of survival rate when there is good control of blood glucose levels, and patients who do not experience bisphosphate osteonecrosis or are not taking bisphosphonates will not be regarded as having a risk factor for low implant-survival rate [31, 32].

In addition to survival rate, titanium (Cluster 3) is considered to have vital research value and great received attention. Since the introduction of Professor Branemark's osseointegration theory, titanium has been considered the best material for implants. Studies have shown that titanium surface roughness and mechanical properties are key to the success of dental implants [24]. Some new surface treatment methods such as discrete crystal deposition, laser ablation, and surface coating with proteins, drugs, or growth factors are also gradually being applied to the clinic [33]. In 1994, Li and other scholars pointed out that the formation of hydroxyapatite is essential for osseointegration between the implant surface and living bone [34]. Nevertheless, the clinical problem of bone resorption was not addressed until decades later. The friction and corrosion resistance of Titanium are also major influencing factors of success rate. Therefore, there should be an effective standard method for testing titanium implants [35].

Sinus floor elevation (Cluster 1) was first reported as a surgical solution to insufficient bone mass in the posterior teeth of the maxilla by Boyne in the 1960s and has since generated attention in the oral implant field. It is well known that the bone mass in the posterior maxillary region is closely related to the initial stability of the implant, which is a key factor influencing the success of the implant. The bone mass in this area is often inadequate to meet the implant needs. Raghoebar et al. concluded in a meta-analysis of patients at 11 research centers who were followed up for at least 5 years that maxillary sinus augmentation floor (MSFA) with lateral window openings could provide stable support for oral implantation [36]. In another 10-year study, Nedir et al. found that when using the alveolar crest approach, even without bone grafting, the effect of maxillary sinus lifting remained ideal, with good implant retention and bone formation in the sinus 1 year after implantation [37], consistently with our findings.

GBR (Cluster 1) was derived from GTR technology from the field of periodontology and is one of the most important technologies for implant surgery. The guided bone regeneration barrier membrane artificially establishes a biological barrier between gum soft tissue and bone defects to prevent fibroblasts and epitheliocytes from growing into bone defect areas ensuring that bone-forming processes in bone defect areas are not disturbed by epithelial tissue [38]. The purpose of GBR is to ensure that the osseointegration process is performed without interference by invasive fibrous soft tissue up until completion.

Different methods of bone regeneration can be used for different degrees of bone defects [39]. In the GBR technique of implant surgery, the choice of barrier membranes is also of great importance in the bone grafting method. By way of illustration, a previous study by Arunjaroensuk et al. comparing the advantages and disadvantages of nonresorbable membranes, resorbable membranes, and collagen-resorbable membranes revealed that resorbable membranes had a similar effect to collagen-resorbable membranes in terms of bone mass enhancement [40]. It is of great significance to guide bone regeneration barrier membrane to reconstruct the morphology and function of periodontal tissue. Hence, it accelerates the development of periodontal therapy and oral implantology. By application of cell occlusive membranes that mechanically exclude nonosteogenic cell populations from the surrounding soft tissues, GBR has become a well-documented and highly successful procedure for bone augmentation [41], which is often used to reconstruct the alveolar ridge when the bone defect occurs in the implant area. The importance of GBR, as a hard tissue augmentation technique, in the furnishing bony support for implant placement has acknowledged diffusely by researchers [42]. Nowadays, some scholars have admitted that GBR represents the gold standard, and it allows obtaining sufficient bone volumes for a correct implant-prosthetic rehabilitation [43, 44]. The close relationship between GBR and implant is consistent with the result in this study. Currently, GBR with titanium mesh has various clinical applications [43], including different clinical procedures. There are various options such as bone graft materials, titanium mesh covering methods, and titanium mesh fixing methods. The GBR with titanium developed some multifarious progress in digitalization and material modification [45]. The combination of GBR and materials science further increases the space for GBR to play in oral implantology. In oral implantology, the application of GBR has been advocated for the promotion of new bone formation and for the preservation of the volume and contour of the alveolar ridge following tooth extraction. Hence, the GBR can play a role in alveolar ridge augmentation before implant placement, immediate implant placement in fresh extraction sockets, and alveolar ridge width and height augmentation in combination with implant placement. Moreover, as an ideal treatment of critical size osseous defects, the GBR can be regarded as an effective therapy for maxillofacial defects and calvarial defects. It is worth mentioning that a series of animal studies have demonstrated that neo-osteogenesis can be predictably achieved via application of the GBR principle [46].

Although many reports have been made analyzing the success rates after onlay bone grafting, data remain scarce on the healing of the soft tissue around the implant [47]. Other reports of bone grafting techniques like bone splitting have revealed higher implant success rates but with a series of postoperative complications, such as fractures of the buccal bone plate and peripheral bone resorption than cannot be ignored. Therefore, to date, GBR is still the best bone grafting technique. In addition, with the development of GBR in recent years, connective tissue transplantation has also become worthy of attention. Previous studies only focused on bone mass, but now, scholars have achieved the goal of increasing bone density and obtained better “soft-hard tissue” and “red-white region” aesthetic effects through connective tissue transplantation without affecting gingival thickness [48, 49]. This further suggests that the prerequisite of dental implant surgery is GBR technology, echoing the results of our study, while other bone grafting techniques remain controversial due to the potential complications surrounding onlay bone grafting and bone splitting.

Through our literature search and analysis using prostheses (Cluster 2) as the keyword, we found that prostheses are directly and closely related to implant survival rate and peri-implantitis. On the one hand, wearing temporary restoration following tooth extraction has a positive clinical effect on preserving the soft tissue around the implant. On the other hand, for the second-stage restoration of the implant, the treatment of the abutment restoration and the cleaning of the adhesive are all important causes of peri-implantitis. Furthermore, compared with traditional denture restorations, implant restorations have unique advantages in terms of functionality, biology, and aesthetics [50, 51].

In the second quadrant, peri-implantitis (Cluster 0) showed a high research focus but poor potential. This is likely related to the continuous development of implant technology, with the gradual standardization and maturity of clinical procedures. Thus, most of the causes of peri-implantitis, including infections, poor oral hygiene, clinical procedures, and even cleaning of the adhesive, are all controllable factors. However, inflammation around the implant is one of the important causes of infection and marginal bone resorption, which in turn affects the survival rate of the implant. Peri-implant inflammation has been included in the classification guide for periodontal disease. Therefore, clinical monitoring and early diagnosis are essential to reduce clinical failure and improve implant survival [52].

In the third quadrant were unpopular or controversial studies like morphology, design, and surface-roughness of implants. Since Parel et al. proposed the theory of “osseointegration” in 1970s [53]; titanium and titanium alloys have become the most popular materials used in dental implant due to their good biocompatibility and mechanical properties, represented by industrial pure titanium TA3 and Ti-6Al-4V alloys with low corrosion resistance and high strength. Although titanium is an active metal, its surface can form TiO_2_, as the main component of the oxidation film, to protect its internal metal further corrosion. However, the oral environment is relatively special. In the process of implant surgery and implant use, implants will be subject to external forces and long-term in the oral cavity, an unstable environment. It increases the chance of abrasion and corrosion, which will cause the degradation of the oral dental implant material [54]. Abrasion and corrosion both can produce metal ions. These ions and particles have a negative effect on a variety of cellular functions in the body [55]. With the popularization of planting technology, the number of young toothless patients accepted implant treatment has increased. The implants will be in the patient's body long term or even contact with tissue and exercise function lifelong. This places high demands on the corrosion resistance and abrasion resistance of implants. The fusion of the surface of a dental implant with the surrounding bone plays a crucial role in the longevity and the function of the implant-supported prosthesis [56]. According to a classical theory, one of the most major causes for implant failure is the implantitis, the destructive inflammation, which occurs surrounding the implant abutment due to the colonization of bacterial [57]. So the type of implant abutment connection may be a key for the success of implant. Biomaterial surfaces may undergo various modifications affecting their physical, chemical, and viscoelastic properties in order to obtain an optimal surface topography, which has been shown to influence osseointegration [58]. As some previous studies have shown, surface modification can produce microrough implant surface, which can accelerate the process of osseointegration. Sandblasting followed by acid etching is regarded as the gold standard technique to create microrough surface. Also, it has been utilized widely in clinic. Moreover, chemical surface modification, such as coating with tricalcium phosphate of titanium and titanium alloy implants [59], leads to higher hydrophilicity and further increases the speed of osseointegration of titanium and titanium-zirconium implants in animals and human [60]. It is still a puzzle that improves the survival rate of implant for researchers and clinicians over the world. For the decades, the researchers focus on the implant interface modification to solve this problem. Even we can draw a conclusion that the success of implants in clinical work is inseparable from the development of materials science. In addition to material science, the survival rate of implant is influenced by other factors including occlusal forces [61]. The correlation between them is also reflected in this study. While Javed et al. questioned the connection between the roughness and basic stability of the implant's surface morphology, no clear conclusion has been reached [62].

In the fourth quadrant, immediate loading (Cluster 2) emerged as a widespread issue of concern. Immediate loading was considered to have a close success rate compared to non-ready-to-load implants, in accordance with the principles of implanting design [63]. However, the risks resulting from failed immediate loading were far higher than those following regular loading [64]. In addition, for some edentulous single implants, immediate loading into the jaw showed poorer survival rates than delayed loading [65]. Hence, although immediate loading has received a high degree of attention, the technology in this field remains immature. Suitable sites for immediate loading should be mainly produced by fresh trauma without any periodontitis or granulation tissue. Even though quite a few clinicians currently perform immediate loading, the indications for immediate implantation are extremely strict and such a procedure is not suitable for all implant patients, sometimes leading to a nonideal survival rate. However, the future implanting trend is that the whole implanting process and results are increasingly rapid and favorable. So new selection criteria are required, and the clinical procedure must be carried out in strict accordance with International Team for Implantology (ITI) standards [66]. More future prospects can be expected, including from immediately successful clinical trials with immediate loading, which aim to solving the issue of denture in edentulous patients [67]. From the prospective of bibliometrics, the immediate-loading and marginal bone loss may be the valuable direction for future researches. Even we can forecast that the future hotspots include the immediate-loading and marginal bone loss based on the bibliometrics arithmetic. It can provide a reference for researchers and institutions to choose topics.

The seven subitems generated by the cocitation analysis in the timeline visualization were used in the dental implant study. Autologous bone (#2) rate was at the core of implanting research and was closely related to risk indicators (#0) and complication rates (#7), which are both direct factors affecting survival. With respect to the study of risk factors, the 2011 Lang article, which determined the baseline for the diagnosis of inflammation around the implant by clinical and imaging data observation, has been indexed 488 times in this field [68]. Jung et al. and Lang et al. have more cocitation links than any other, indicating that these two articles may be of key significance in the research and analysis of implant survival and osteointegration. Jung et al. also reported that single dental implants and the corresponding single crowns have higher implant survival rates within 10 years. However, technical, biological, and aesthetic complications do occur from time to time [69]. Lang et al. evaluated the rate and extent of osseointegration during the early stages of bone healing with moderately rough implants that were chemically modified to be either hydrophilic (SLActive) or hydrophobic (SLA) [70]. In recent years, the focus of research on dental implants has thus gradually shifted from survival to complication rate. This change may be due to the continuous improvement of implant survival, resulting in the focus of the study evolving into implant complications.

However, there are still some limitations in this study. Because collaborative cluster analysis of high-frequency MeSH terms is a new analysis method, researchers will have a certain degree of bias when selecting vocabulary. Due to functional limitations in the PubMed and Web of Science database lacking other database retrieval results, the data set in this study may be incomplete. In addition, due to the inconsistent quality of articles, errors in some research results are inevitable. At present, the visualization software we use, such as Ucinet 6, VOSviewer, SCIMAT, and other processing documents, often can only process one database in one time, which has limitations of biased results. The results of analyzing multiple databases will be more objective and accurate.

## 5. Conclusions

We used different scientometric tools to perform a coword analysis and cocitation analysis on the literature of dental implants and identified 4 clusters and 7 subcategories of research subjects. To this date, implant survival rates and second-stage repair remain central hot topics in the field of implant research. In addition, sinus floor elevation and GBR are the primary auxiliary technologies first considered. While immediate implantation is still not a mainstream procedure in the clinic and indications need to be strictly controlled, research and technical breakthroughs in the future may turn it into a big research hotspot, making immediate implantation a conventional surgical method.

## Figures and Tables

**Table 1:** High-frequency keywords in dental implant research.

No.	Keyword	Frequency *N* (%)		Cumulative percentage (%)
1	Dental implant	6386	(9.70)	9.70
2	Osseointegration	985	(1.50)	11.20
3	Titanium	726	(1.10)	12.30
4	Peri-implantitis	532	(0.81)	13.11
5	Bone regeneration	362	(0.55)	13.66
6	CBCT	355	(0.54)	14.20
7	Sinus floor elevation	353	(0.54)	14.74
8	Immediate loading	352	(0.53)	15.27
9	Zirconia	321	(0.49)	15.76
10	Finite element analysis	305	(0.46)	16.22
11	Hydroxyapatite	199	(0.30)	16.53
12	Biomaterials	196	(0.30)	16.82
13	Bone substitute	191	(0.29)	17.11
14	Systematic review	190	(0.29)	17.40
15	Biocompatibility	188	(0.29)	17.69
16	Bone graft	186	(0.28)	17.97
17	Bone	182	(0.28)	18.25
18	Alveolar bone loss	167	(0.25)	18.50
19	Complication	167	(0.25)	18.75
20	Short dental implant	166	(0.25)	19.01
21	Surface modification	164	(0.25)	19.26
22	Marginal bone loss	161	(0.24)	19.50
23	Guided bone regeneration	147	(0.22)	19.72
24	Bone loss	142	(0.22)	19.94
25	Maxillary sinus	139	(0.21)	20.15
26	Mandible	136	(0.21)	20.36
27	Implant survival	126	(0.19)	20.55
28	Histology	125	(0.19)	20.74
29	Meta-analysis	121	(0.18)	20.92
30	Periodontitis	121	(0.18)	21.11
31	Resonance frequency analysis	117	(0.18)	21.28
32	Biomechanics	114	(0.17)	21.46
33	Implant stability	114	(0.17)	21.63
34	Bone augmentation	111	(0.17)	21.80
35	Maxilla	109	(0.17)	21.96
36	Survival	109	(0.17)	22.13
37	Survival rate	103	(0.16)	22.29
38	Bone resorption	101	(0.15)	22.44
39	Overdenture	100	(0.15)	22.59
40	Patient satisfaction	98	(0.15)	22.74
41	Tissue engineering	95	(0.14)	22.89
42	Biofilm	93	(0.14)	23.03
43	Dental prosthesis	93	(0.14)	23.17
44	Implant failure	91	(0.14)	23.31
45	Inflammation	89	(0.14)	23.44
46	Bone density	84	(0.13)	23.57
47	Prosthodontics	84	(0.13)	23.70
48	Implant surface	84	(0.13)	23.82
49	Platform switching	82	(0.12)	23.95
50	Alveolar ridge augmentation	81	(0.12)	24.07
51	Primary stability	80	(0.12)	24.19
52	Bone remodeling	79	(0.12)	24.31
53	CAD/CAM	78	(0.12)	24.43
54	Insertion torque	78	(0.12)	24.55
55	Corrosion	77	(0.12)	24.67
56	Clinical research	75	(0.11)	24.78
57	Tooth extraction	75	(0.11)	24.90
58	Histomorphometry	73	(0.11)	25.01
59	Osteoporosis	73	(0.11)	25.12
60	Quality of life	71	(0.11)	25.23
61	Animal study	70	(0.11)	25.33
62	Esthetics	67	(0.10)	25.43
63	Edentulous mandible	67	(0.10)	25.54
64	Implant-supported dental prosthesis	67	(0.10)	25.64
65	Accuracy	66	(0.10)	25.74
66	Bioactivity	65	(0.10)	25.84
67	Risk factors	65	(0.10)	25.94
68	Smoking	62	(0.09)	26.03
69	Peri-implant mucositis	61	(0.09)	26.12
70	Soft tissue	61	(0.09)	26.21
71	Implant design	61	(0.09)	26.31
72	Early loading	60	(0.09)	26 .40

**Table 2:** Binary matrix of high-frequency keywords and dental implant articles.

No.	Keyword	Paper ID				
		000001	000004	000007	…	015770
1	Dental implant	1	0	0	…	1
2	Osseointegration	0	0	0	…	0
3	Titanium	0	0	0	…	0
4	Peri-implantitis	0	0	0	…	0
5	Bone regeneration	1	0	0	…	0
…	…	…	…	…	…	…
71	Implant design	0	0	0	…	0
72	Early loading	0	0	0	…	0

**Table 3:** Coword matrix of high-frequency keywords in dental implant articles.

No.	Keyword	Dental implant	Osseointegration	…	Malocclusion
1	Dental implant	6386	619	…	45
2	Osseointegration	619	985	…	3
3	Titanium	342	96	…	0
4	Peri-implantitis	327	14	…	0
…	…	…	…	…	…
72	Early loading	45	3	…	60

## Data Availability

All data generated or used during the study appear in the submitted article.
